# Mechanically Derived Tissue Stromal Vascular Fraction Acts Anti-inflammatory on TNF Alpha-Stimulated Chondrocytes In Vitro

**DOI:** 10.3390/bioengineering9080345

**Published:** 2022-07-27

**Authors:** Joeri van Boxtel, Lucienne A. Vonk, Hieronymus P. Stevens, Joris A. van Dongen

**Affiliations:** 1 Department of Plastic Surgery, University Medical Center Groningen, University of Groningen, 9713 GZ Groningen, The Netherlands; jorisavandongen@gmail.com; 2 Department of Plastic Surgery, Catharina Hospital, 5623 EJ Eindhoven, The Netherlands; 3 Department of Orthopaedics, University Medical Center Utrecht, Utrecht University, 3584 CX Utrecht, The Netherlands; l.vonk@codon.de; 4 CO.DON AG, 14513 Teltow, Germany; 5 PRSkliniek, 3137 XB Vlaardingen, The Netherlands; info@prskliniek.nl; 6 Department of Pathology & Medical Biology, University Medical Center Groningen, University of Groningen, 9713 GZ Groningen, The Netherlands

**Keywords:** t-SVF, stromal vascular fraction, anti-inflammatory, joint, osteoarthritis, FAT

## Abstract

Enzymatically isolated stromal vascular fraction (SVF) has already shown to be effective as a treatment for osteoarthritis (OA). Yet, the use of enzymes for clinical purpose is highly regulated in many countries. Mechanical preparation of SVF results in a tissue-like SVF (tSVF) containing intact cell–cell connections including extracellular matrix (ECM) and is therefore less regulated. The purpose of this study was to investigate the immunomodulatory and pro-regenerative effect of tSVF on TNFα-stimulated chondrocytes in vitro. tSVF was mechanically derived using the Fractionation of Adipose Tissue (FAT) procedure. Characterization of tSVF was performed, e.g., cellular composition based on CD marker expression, colony forming unit and differentiation capacity after enzymatic dissociation (from heron referred to as tSVF-derived cells). Different co-cultures of tSVF-derived cells and TNFα-stimulated chondrocytes were analysed based on the production of sulphated glycosaminoglycans and the anti-inflammatory response of chondrocytes. Characterization of tSVF-derived cells mainly contained ASCs, endothelial cells, leukocytes and supra-adventitial cells. tSVF-derived cells were able to form colonies and differentiate into multiple cell lineages. Co-cultures with chondrocytes resulted in a shift of the ratio between tSVF cells: chondrocytes, in favor of chondrocytes alone (*p* < 0.05), and IL-1β and COX2 gene expression was upregulated in TNFα-treated chondrocytes. After treatment with (a conditioned medium of) tSVF-derived cells, IL-1β and COX2 gene expression was significantly reduced (*p* < 0.01). These results suggest mechanically derived tSVF stimulates chondrocyte proliferation while preserving the function of chondrocytes. Moreover, tSVF suppresses TNFα-stimulated chondrocyte inflammation in vitro. This pro-regenerative and anti-inflammatory effect shows the potential of tSVF as a treatment for osteoarthritis.

## 1. Introduction

Autologous adipose tissue transplantation is frequently used for a variety of different clinical indications, such as dermal scarring, fat atrophy, body contouring, wound healing, burn wounds, osteoarthritis and perianal fistulas [1,2,3,4,5,6]. The therapeutic efficacy of adipose tissue is ascribed to the stromal vascular fraction (SVF) containing a heterogeneous mixture of non-adipocyte cell types e.g., immune cells, fibroblasts, endothelial cells, pericytes and adipose derived stromal cells (ASCs) [7,8]. ASCs reside in SVF as progenitor cell types attached around vessels as pericytes or supra-adventitial cells [9,10]. In vitro, ASCs secrete a plethora of growth factors, cytokines, chemokines, matrix proteases and extracellular vesicles which stimulate different regenerative processes such as angiogenesis, fibroblast migration and proliferation, matrix remodeling as well as immune modulation [11,12,13]. These trophic effects illustrate the regenerative potential of ASCs as a source for cell-based therapy to repair different types of damaged tissue [14].

Nowadays, SVF is often mechanically derived instead of enzymatically isolated because the use of enzymes for medicinal products is highly regulated in many countries. Mechanical preparation of SVF results in a tissue-like SVF (tSVF) containing SVF cells and intact cell–cell communications including ECM. Enzymatically isolated SVF yields a single cell suspension of SVF (cellular SVF or cSVF). ECM acts as a pro-regenerative scaffold to bind and release growth factors, matrix metalloproteinases (MMP), proteins and cytokines in a controlled way [15,16]. Several growth factors or enzymes, such as transforming growth factor-beta (TGF-β), fibroblast growth factor (FGF) and matrix metalloproteinase 13 (MMP-13), are involved in cartilage homeostasis [17,18,19]. A disbalance between these growth factors and enzymes plays an important role in cartilage degradation, synovitis and osteophyte growth and, thus, osteoarthritis (OA) [17,18,19]. Osteoarthritis is chronic progressive disease, wherein damaged joints lead to pain, stiffness and disability [20]. ASCs might decrease these clinical symptoms by influencing growth factor stimulated processes involving cartilage degeneration such as blocking TGF-β1-induced fibrosis and reducing collagenase 3 (MMP-13) expression in vitro [21,22]. Furthermore, ASCs can induce immune suppressive effects in OA by exciting macrophages in the synovium to produce interleukin (IL)-10 or induce a switch to M2 macrophages via secretion of prostaglandin-E_2_ (PGE2) [23]. This could beneficially contribute to the treatment of OA. Hence, tSVF as a source for ASC-cell-based therapy potentially harbors more therapeutic capacity than ASCs alone in the treatment of OA.

Multiple studies have already shown safety and efficacy e.g., improved functionality, less instability and reduction of pain of using ASCs and cSVF as a treatment of OA in humans and animals [24,25,26,27]. More recently, a case series with tSVF as a treatment for knee OA in human subjects demonstrated tSVF is also safe to use [28]. The purpose of this study was to investigate the immunomodulatory and pro-regenerative effect of mechanically derived tSVF on TNFα-stimulated chondrocytes in vitro.

## 2. Material and Methods

### 2.1. Lipoharvesting and the FAT Procedure

Lipoaspirate was collected from healthy female patients between 18 and 65 years of age with a mean BMI of 27 (18.4–34, *n* = 11). The FAT procedure was performed in a standardized way as published earlier (Appendix A) [16].

### 2.2. Enzymatic Dissociation of tSVF

Samples of tSVF were washed with phosphate buffered saline (PBS) three times. After washing, 0.1% collagenase A in PBS/1% bovine serum albumin (BSA) was added as dissociation medium. Samples were stirred for 1.5 h in a 37 °C water bath. Cells were resuspended in erythrocyte lysisbuffer (ThermoFisher, Waltham, MA, USA) and incubated for 5 min. Samples were centrifuged at 8 °C, 600× *g* for 10 min. and resuspended in expansion medium consisting of Dulbecco’s Modified Eagle’s Medium (DMEM; Gibco, Paisley, UK) supplemented with 10% fetal bovine serum (FBS; HyClone, Logan, UT, USA) and 1% Penicillin/Streptomycin). Viable cells were counted with trypan blue in a Bürker Turk counting chamber. Cells collected from enzymatically processed tSVF samples are henceforth referred to as ‘tSVF-derived cells’.

### 2.3. Flow Cytometry to Determine Cell Composition of tSVF

tSVF-derived cells i.e., from the one-hole re-usable fractionator (*n* = 11), were analysed for CD surface marker expression using flow cytometry. Cells were labelled with the following anti-human monoclonal antibodies: CD31, CD34, CD90, CD105, CD146 (Miltenyi Biotec, Bergisch Gladbach, Germany) and CD45 (Biolegend, San Diego, CA, USA) as well as 7-Amino Actinomycin D (Invitrogen, molecular probes, Eugene, OR, USA) to stain for dead cells. Cells were mixed well with the antibodies in FACS buffer (5 mM ethylenediaminetetraacetid acid (EDTA), 1% BSA in PBS) and incubated on ice and in the dark for 30 min. Stainings with a single antibody and fluorescence minus one (FMO) were used as controls. A BD FACSCanto II system (BD Biosciences, San Diego, CA, USA) was used to analyse the samples.

### 2.4. Colony Formation Unit Capacity of ASCs

Ten thousand viable cells enzymatically processed from tSVF passage 0 as well as thousand cells from passage 1 and 2 (*n* = 11 and six technical replicates) were seeded and cultured for twelve days to assess the colony forming capacity. Afterwards, cells were fixed in 4% formalin and stained with 5% Crystal Violet (Sigma-Aldrich, St. Louis, MO, USA). Colony frequency was calculated as the mean number of colonies/total seeded cells × 100%.

### 2.5. Differentiation Capacity of tSVF and ASCs

tSVF-derived cells from passage 0, 1, 2, 3 (*n* = 11) were collected and used for adipogenic, osteogenic and chondrogenic differentiation assays.

For adipogenic differentiation, the cells were seeded at 960,000 for P0 and 240,000 for P1, P2 and P3 cells per well of a 24-well plate. The P0 were cultured for 72 h and P1, 2 and P3 cells for 24 h before the medium was changed to an adipogenic differentiation medium that consisted of alpha-MEM supplemented with 10% FBS, 500 µM 3-isobutyl-1-methylxanthine (IBMX), 1 µM dexamethasone, 0.2 mM indomethacin, 1.72 µM insulin and pen/strep. The cells were cultured in an adipogenic medium for 3 weeks and the medium was changed three times a week. After culture, the cells were fixed in formalin, washed with PBS followed by 60% isopropanol, stained with Oil red O, washed with 60% isopropanol and washed with PBS, whereafter they were microphotographed.

For osteogenic differentiation, the cells were seeded at a density of 128,000 viable nucleated cells for passage 0 and 32,000 viable nucleated cells for passages 1, 2 and 3 per well of a 12-well plate in an expansion medium. The P0 were cultured for 72 h and P1, P2 and P3 cells for 24 h before the medium was changed to osteogenic differentiation medium. The osteogenic differentiation medium consisted of alpha-MEM (Gibco) supplemented with 10% FBS, 10 nM dexamethasone, 0.2 mM ascorbic acid-2-phosphate, 10 mM of β-glycerophosphate and pen/strep. The cells were cultured in an osteogenic medium for 3 weeks and the medium was changed three times a week. After 3 weeks, the cells were fixed with formalin and washed with PBS and stained for mineralization with 3% Alizarin Red S and washed 6 times with PBS, whereafter microphotographs were taken.

For chondrogenic differentiation, 1 million P0 or 250,000 P1, P2 and P3 viable nucleated cells were seeded in a 15 mL falcon tube that was centrifuged for 5 min at 300× *g* to pellet the cells. After 3 days, the culture medium was replaced by a chondrogenic medium consisting of DMEM supplemented with 1% ITS+ premix (354352; BD Bioscienes), 10−7 M dexamethasone (D8893; Sigma), 0.2 mM L-ascorbic acid 2-phosphate, 1% Pen/Strep and 10 ng/mL TGF-β2 (302-B2; R&D Systems). The cells were cultured for 4 weeks in a chondrogenic medium and the medium was changed 3 times per week. After 28 days of culture, pellets were formalin-fixed and paraffin embedded. Subsequently, 5 μm sections were stained with 0.125% safranin-O (Merck, counterstained with Weigert’s hematoxylin (Klinipath) and 0.4% fast green (Merck)) to stain deposited proteoglycans.

### 2.6. Enzymatic Isolation of Chondrocytes

Chondrocytes were isolated from donor cartilage of various human patients after total knee arthroplasty due the end-stage OA. The use of the tissue for this purpose was allowed by the local ethical committee of the UMC Utrecht (TC-Bio protocolnumber 15-092). Cartilage was minced and tissue fragments were subjected to sequential treatments, first with DMEM supplemented with 1% FBS, 100 U/mL of penicillin, 100 mg/mL of streptomycin and 2.5% (*w*/*v*) of pronase E (Sigma, St. Louis, MO, USA) for 1 h, then with DMEM supplemented with 25% FBS, 100 U/mL of penicillin, 100 mg/mL of streptomycin and 0.125% (*w*/*v*) of collagenase (CLS-2; Worthington, Lakewood, NJ, USA) for 16 h at 37 °C. Cells were filtered through a 70-mm cell strainer (BD Biosciences, San Diego, CA, USA) and washed. Chondrocytes were expanded to passage 2 in T175 flasks in an expansion medium.

### 2.7. Functional Analysis of Co-culture of tSVF-Derived Cells and Chondrocytes

Freshly isolated tSVF-derived cells and passage 2 chondrocytes were cultured in various ratios in pellets. A total of 250,000 cells, consisting of 0, 25, 50, 75 and 100% nucleated tSVF-derived cells and 100, 75, 50, 25 and 0% chondrocytes, were cultured in 1 mL DMEM (Gibco) supplemented with 2% insulin-transferrin-selenium (ITS)-X (Invitrogen), 50 µg/mL ascorbate-2-phosphate (Sigma), 2% human serum albumin (Sanquin) and penicillin/streptomycin (100 U/mL, 100 µg/mL), which was refreshed every 2–3 days at 37 °C at 5% CO_2_ in 15 mL Falcon tubes (ThermoFisher). Immediately after pelleting and after 21 days of culture, RNA was isolated from 3 pellets for RT real-time PCR to investigate the ratio between the cells. After 28 days, 3 pellets were papain digested for biochemical analyses.

#### 2.7.1. Cell Ratio Analysis of Chondrocytes and tSVF-Derived Cells Co-cultures

Co-cultures of tSVF-derived cells from female donors and chondrocytes from male donors (*n* = 3 in triplicates) in various ratios were cultured for 21 days. After 21 days, the expression of the Y-chromosomal gene lysine demethylase 5D (KDM5D) gene was measured. In addition, 18S was used as the housekeeping gene. Total RNA was isolated using Trizol (Invitrogen) as described by the manufacturer. Total RNA (500 ng) was reverse transcribed using the high-capacity cDNA Reverse Transcription kit (Thermo Fisher Scientific, Waltham, MA, USA). Real-time polymerase chain reactions were performed using iScript universal SYBR Green (Biorad) in a LightCycler 96 (Roche). Primers used are shown in Table 1.

#### 2.7.2. Sulphated Glycosaminoglycans Analysis

After 28 days of culture, co-cultures of tSVF-derived cells and chondrocytes (*n* = 3 in triplicates) were digested at 60 °C for 18 h in a papain enzyme solution consisting of 5 mM L-cysteine, 50 mM Na2 EDTA, 0.1 M NaAc and pH 5.53 with 2% (*v*/*v*) papain (Sigma). To measure the concentration of sulphated glycosaminoglycans (GAGs), a dimethylene blue (DMMB) spectrophotometric analysis was performed. The papain digests were 1:10 diluted and mixed with the DMMB solution and absorbance was read at 540 nm and 595 nm. Known concentrations of chondroitin sulphate C (Sigma) were used as a reference. To correct for the number of cells, the DNA amount in the papain digests was measured. Papain digests were diluted 1:20 and Quant-iT Picogreen (Invitrogen) reagent was added. This was incubated for 5 min. at an ambient temperature in the dark, whereafter the fluorescence was measured at 480 nm excitation and 520 nm emission. Known concentrations of lambda DNA were used as a standard.

#### 2.7.3. In Vitro Inflammation Assay

Chondrocytes (*n* = 3 in triplicates) were cultured in monolayer at 37 °C and 5% CO_2_ at a seeding density of 25,000 cells/cm^2^ in an expansion medium. After pre-incubation for 24 h, cells were treated with 10 ng/mL tumor necrosis factor (TNF)-alpha (Immunotools). After 24 h of treatment, the cells were washed and the medium was refreshed with either control medium, conditioned medium (CM) from cultured chondrocytes and cultured tSVF-derived cells or chondrocytes or tSVF-derived cells were added. After 24 h, RNA was isolated from the monolayers and gene expression of interleukin (IL)-1β and cyclooxygenase-2 (COX2) were measured as described in 2.7.1. Again, 18S was used as the housekeeping gene. Primers used are shown in Table 1.

### 2.8. Statistical Analysis

Descriptive statistics were used to evaluate the number of cells in tSVF, cell composition of tSVF, colony forming units as well as the number of cells after co-culture of chondrocytes and tSVF-derived cells, the amount of sGAG and the expression of IL-1β and COX2. Data were expressed as mean with standard deviation. A two-tailed paired *t*-test was performed to analyze the number of cells after co-culture of chondrocytes and tSVF-derived cells. A two-tailed unpaired t test was performed to analyze the amount of sGAG and the expression of IL-1β and COX2. All data was analyzed using Graphpad Prism, version 5.01 (Graph Pad Software Inc., Los Angeles, CA, USA).

## 3. Results

### 3.1. Characterization of tSVF

**Figure 1:**
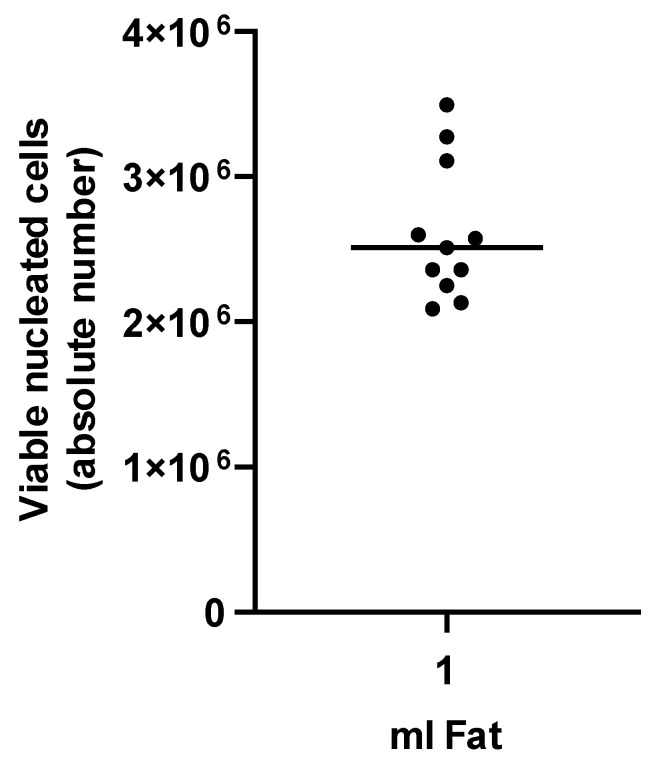
Number of viable nucleated cells per mL tissue-like stromal vascular fraction (tSVF). Each dot represents the value of one donor.

**Figure 2:**
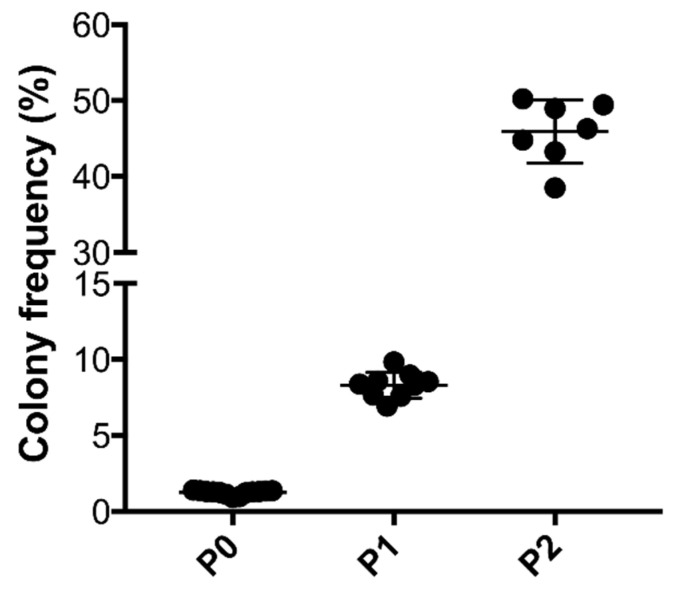
Colony-forming unit assay of tissue-like stromal vascular fraction (tSVF) at P0 (10,000 cells seeded), P1 (1000 cells seeded) and P2 (1000 cells seeded) to examine cell colony formation. The number of violet/blue-stained colonies is expressed as a percentage of total seeded nucleated cells. P = passage.

**Figure 3:**
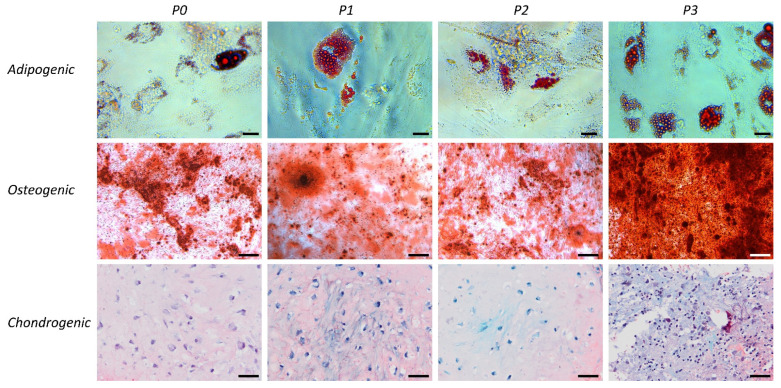
Representative bright-field microscope images of tissue-like stromal vascular fraction (tSVF) at P0, P1, P2 and P3 differentiated into the adipogenic (upper, 40× magnification), osteogenic (mid, 10× magnification) and chondrogenic (lower, 10× magnification) lineages as shown by stainings with oil red O, alizarin red and safranin-O, respectively. The scale bar of the adipogenic images is 10 micrometer and 50 micrometer, respectively, for the osteogenic and chondrogenic images. P = passage.

Enzymatic isolation of tSVF yielded a mean number of viable cells of 2.67 × 10^6^ ± 4.63 × 10^5^ per 1 mL (Figure 1). Composition of tSVF contained a mean number of 48.87% ± 17.59% of ASCs (CD45−; CD90+; CD105+), 39.55% ± 32.38% of endothelial cells (CD31+; CD34+), 3.42% ± 3.18% of leukocytes (CD45+; CD34−), 0.23% ± 0.19% of pericytes (CD34+/−; CD31−; CD146+), 0.32% ± 0.39% of hematopoietic stem cell-like cells (CD45+; CD34+) and 5.95% ± 8.54% of supra-adventitial cells (CD34bright; CD31−, CD146−) (Table 2). After culture, cells present in tSVF were cultured and characterized based on their ability to form colonies which increased each passage (Figure 2). Moreover, primary freshly isolated and plastic-adherence expanded tSVF-derived cells were able to differentiate into adipogenic, osteogenic and chondrogenic cell lineages (Figure 3).

#### 3.1.1. Cell Ratio Analysis of Chondrocytes and tSVF-Derived Cells in Co-Culture

**Figure 4:**
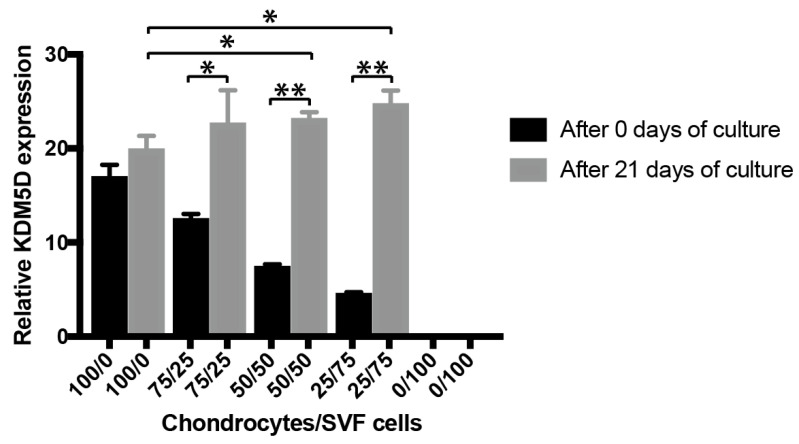
Relative lysine demethylase 5D (KDM5D, y-chromosomal) gene expression to 18S of male chondrocytes after 0 days and 21 days of culture. Results are expressed as mean ± SD and analysed with an unpaired *t*-test when different pellets of co-cultures were compared. A paired *t*-test was used when the same co-culture was analysed at different timepoints. * *p* ≤ 0.05. ** *p* ≤ 0.01.

Chondrocytes co-cultured with tSVF-derived cells in various ratios, i.e., 75%/25%, 50%/50% and 25%/75% (chondrocytes/tSVF-derived cells) resulted in an increase of the ratio chondrocytes/tSVF-derived cells after 21 days compared to the baseline (*p* < 0.05, *p* < 0.01, *p* < 0.01, respectively, Figure 4). The total number of chondrocytes was significantly higher when co-cultures of chondrocytes and tSVF-derived cells, i.e., 75%/25% and 50%/50% were compared with cultures of chondrocytes alone (*p* < 0.05, Figure 4).

#### 3.1.2. Sulphated GAGs Analysis

**Figure 5:**
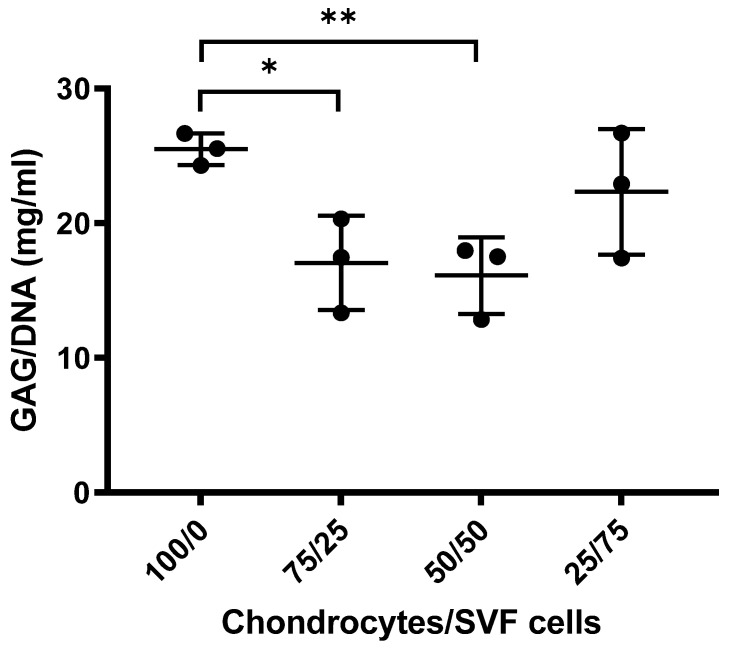
Sulphated glycosaminoglycan (GAG) deposition normalized for DNA content of pellets from co-cultures of chondrocytes and tSVF-derived cells after 28 days of culture at various ratios. Results are expressed as mean ± SD and analysed with an unpaired *t*-test. * *p* ≤ 0.05. ** *p* ≤ 0.01.

No significant difference was observed when mono-cultures of chondrocytes were compared with co-cultures of 25% chondrocytes and 75% tSVF-derived cells (*p* > 0.05, Figure 5). Significantly, more sulphated GAGs were present in mono-cultures of chondrocytes in comparison with co-cultures of chondrocytes and tSVF-derived cells at 75%/25% and 50%/50% after 28 days, respectively (*p* < 0.05, *p* < 0.01, Figure 5).

#### 3.1.3. In Vitro Inflammation Assay

**Figure 6:**
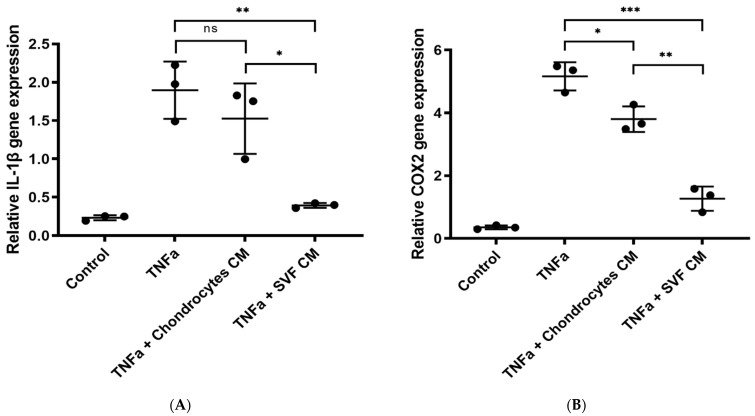
(**A**) Relative gene expression of interleukin (IL)-β (**A**) and cyclooxygenase 2 (COX2 (prostaglandin-endoperoxide synthase 2 (PTGS2)), (**B**) by tumor necrosis factor alpha (TNFα)-stimulated chondrocytes and subsequently treated with conditioned medium (CM) of chondrocytes or tissue-like stromal vascular fraction (tSVF) for 24h. Results are expressed as mean ± SD and analysed with an unpaired *t*-test. ns = not significant. * *p* ≤ 0.05. ** *p* ≤ 0.01. *** *p* ≤ 0.001. Control = chondrocytes without TNFα treatment.

**Figure 7:**
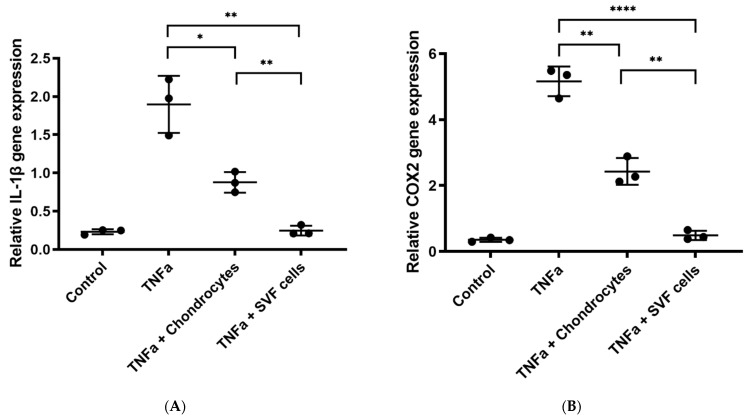
(**A**) Relative gene expression of interleukin (IL)-1β (**A**) and cyclooxygenase 2 (COX2 (prostaglandin-endoperoxide synthase 2 (PTGS2)), (**B**) by tumor necrosis factor alpha (TNFα)-stimulated chondrocytes and subsequently treated with chondrocytes or tissue-like stromal vascular fraction (tSVF) cells for 24h. Results are expressed as mean ± SD and analysed with an unpaired *t*-test. * *p* ≤ 0.05. ** *p* ≤ 0.01. **** *p* ≤ 0.0001. Control = chondrocytes without TNFα treatment.

A significant downregulation of IL-1β gene expression was observed in TNFα-treated chondrocytes after the addition of CM from tSVF-derived cells (*p* < 0.01, Figure 6A), but not after the addition of CM from chondrocytes (Figure 6A). COX2 gene expression was significantly downregulated in TNFα-treated chondrocytes after the addition of CM from chondrocytes and CM from tSVF-derived cells (*p* < 0.05, *p* < 0.01, Figure 6B). Both IL-1β and COX2 gene expression was downregulated in TNFα-treated chondrocytes after addition of tSVF-derived cells or chondrocytes (*p* < 0.01, *p* < 0.0001, Figure 7), but the gene expressions were more reduced by the addition of tSVF-derived cells compared to chondrocytes (*p* < 0.01, Figure 7).

## 4. Discussion

This study demonstrates that enzymatically processed cells from tSVF—which was mechanically derived by means of the FAT procedure—have a pro-regenerative effect and an anti-inflammatory effect on TNFα-stimulated chondrocytes in vitro. Co-cultures of tSVF-derived cells and chondrocytes resulted over time in a significant higher ratio of chondrocytes in various co-cultures with tSVF-derived cells as compared to cultures of chondrocytes alone. A pro-regenerative effect of tSVF is postulated as it enables regeneration of functional cartilage by stimulating GAGs formation in vitro. Downregulation of IL-1β and COX2 gene expression due to addition of tSVF to TNFα stimulated chondrocytes in vitro suggests an anti-inflammatory effect. These aforementioned inflammatory processes are involved in osteoarthritis and can be influenced by the heterogenous cell population of tSVF. Hence, tSVF is a potential effective therapy for the treatment of OA.

Elevated levels of COX2 and IL-1*β* are found in the chronic inflammatory state of joints [29,30,31]. Overexpression of COX2 in osteoarthritis results in an increased production of matrix metalloproteinases (MMPs), reduction of collagen synthesis and stimulation of chondrocyte apoptosis [29]. All of these processes contribute to cartilage degradation, leading to more inflammation. This vicious circle of cartilage degradation and inflammation is enhanced by PGE2, a pro-inflammatory mediator produced after stimulation with COX2 and IL-1β. Eventually, cartilage degradation will result in pain. Thus far, several COX inhibitors, e.g., NSAIDs and selective COX2 inhibitors, have been used to treat clinical symptoms of osteoarthritis by blocking synthesis of COX1 and COX2 [32,33]. Although these oral medicaments seem beneficial, there is a serious risk of systemic complications, e.g., gastro-intestinal ulceration or bleeding and cardiotoxicity [32,33]. Comparable to COX2, IL-1β is a major contributor to inflammatory reactions and catabolic effects to articular cartilage as well. In osteoarthritic joints, elevated levels of IL-1β are present in synovial fluid in cartilage and the subchondral bone layer [30,34]. IL-1β inhibits the ability of chondrocytes to repair cartilage by blocking synthesis of type II collagen and aggrecan in ECM. IL-1β seems to have a direct adverse effect on chondrocytes as it stimulates the synthesis of MMPs, mainly MMP-1, -3 and -13, which have a deteriorating effect on cartilage [34,35]. Indirectly, IL-1β also degrades cartilage ECM by stimulating the production of the aggrecan molecule proteolytic enzyme a disintegrin and metalloproteinase with thrombospondin motifs (ADAMTS) metalloproteinases, especially ADAMTS-4 [34,36]. Considering the downregulation of IL-1β and COX2 gene expression by tSVF as shown in this current study, local treatment of OA by autologous tSVF might be effective and substantially reduces the risk of systemic complications [28]. Moreover, ASCs have shown to secrete high levels of tissue inhibitors of metalloproteinases TIMP-1 and -2, which block the degrading effect of MMPs on cartilage [23]. The effects of TIMPs as well as IL-1β, COX2 and other pro-inflammatory signaling molecules might be reversed by tSVF and could possibly slow down the progression of osteoarthritis in the affected joints, thereby reducing pain.

The change of ratio between cells in co-culture has been shown before in co-cultures between MSCs from various sources and chondrocytes [37,38,39,40]. Moreover, there is clinical evidence that co-implantation of allogeneic MSCs and chondrons leads to effective repair of cartilage defects, whereas the MSCs disappear and do not engraft the repair tissue [41,42]. It is likely that the tSVF-derived cells provide a similar paracrine stimulation to the chondrocytes in the present study. However, there seem to be ratios that are less effective, as has also been shown previously in co-cultures between culture expanded bone marrow MSCs with chondrocytes and with chondrons [43]. A possible explanation is that chondrocytes either proliferate or deposit ECM, but these two activities are not performed effectively together. In this study, there is no difference in the sulphated GAG deposition between monocultures of 100% chondrocytes and a co-culture of 25% chondrocytes with 75% tSVF-derived cells, whereas the co-cultures of 50%/50% and 75%/25% chondrocytes/tSVF deposited significantly fewer GAGs. In the cultures with 50% and 25% tSVF-derived cells, the paracrine signaling by the tSVFs might have been less strong compared to the co-culture with 75% tSVF cells, leading to a prolonged proliferation phase and therefore a delayed ECM deposition phase.

Multiple recently published studies use cSVF as a treatment of osteoarthritis in vitro [44,45,46,47]. In these studies, however, cSVF is obtained enzymatically by time-consuming isolation procedures. These enzymatic procedures disrupt all cell–cell interactions including ECM [48]. Although several studies reported on the therapeutic effect of cSVF on osteoarthritis, the regenerative role of ECM is often underestimated. In tSVF, the ECM is preserved by holding stromal cells, e.g., ASCs in their local niche. In this way, stromal cells might have a higher retention rate after injection and thus probably a prolonged regenerative effect. In addition to having a cell retaining function, ECM influences cells in a complex and mechanical behavioral way [49]. A time-dependent response of cells to loading or deformation, called viscoelasticity, has become widely accepted as a concept wherein mechanical properties of ECM stiffness have an effect on cell proliferation and differentiation. ECM stiffness seems to regulate developmental, homeostatic and regenerative processes [49]. Another important function of ECM is the binding of a plethora of factors and ensuring controlled slow release of these different factors over time [15]. A natural slow release scaffold might contribute to a prolonged regenerative effect of tSVF as compared to cSVF. A limitation of this study is the use of a two-dimensional culture system with a single layer of culture chondrocytes. It is well-known that cells behave significantly differently in a two-dimensional culture system as compared to a three-dimensional culture system. A three-dimensional culture system mimics processes in vivo more accurately [50]. In a two-dimensional culture system, the role of synovial inflammation and subchondral bone remodeling and their interaction with chondrocytes cannot be investigated [50]. In 2013, a three-dimensional osteochondral system to mimic the pathogenesis of osteoarthritis was developed which involved mechanical injury, pro-inflammatory cytokine influence and cartilage degeneration [51]. This might be of importance in the translation of results from in vitro to in vivo. To date, the complex multifactorial pathophysiology of osteoarthritis is not yet fully addressed. This also leads to the second limitation that the in vitro stimulation with TNFα does not perfectly resemble the inflammatory components of osteoarthritis. The method is a well-established and frequently used way to invoke an inflammatory response in chondrocytes, mediated by nuclear factor kappa B (NF-κB), which can be measured by multiple outcomes such as the increase of (gene) expression of IL-1β, COX2, PGE-2, MMPs and nuclear translocation of NF-κB [52,53]. A third limitation of this study is that it predominantly observes the effect of tSVF on chondrocytes, whereas subchondral and synovial processes are known to play an important role in OA as well. However, both chondrocytes and synovial tissue cells produce pro-inflammatory cytokines with similar underlying mechanisms [54]. Therefore, it is likely that synovial cells will respond the same in terms of downregulation of inflammatory factors in response to tSVF. Moreover, chondrocytes are the cells that produce cartilage tissue and the co-culture of chondrocytes with tSVF-derived cells resulted in the proliferation of chondrocytes and increased GAG deposition. Performing a well-designed in vivo study could diminish these limitations as it would allow to study the effects in an OA environment where the effects on all cells and tissues that are involved in OA can be studied.

The results of this study show that mechanically derived tSVF is able to promote chondrocyte proliferation and suppress chondrocyte inflammation in vitro. As a result, tSVF has shown to have an anti-inflammatory as well as a pro-regenerative effect on TNFα-stimulated chondrocytes. Consequently, tSVF as a treatment for osteoarthritis seems to be very promising. To further elucidate the beneficial effect of tSVF on osteoarthritis clinically, prospective randomized clinical trials are necessary.

## Figures and Tables

**Table 1:** Primers used for cell number analysis of chondrocytes and tSVF-derived cells co-cultures and in vitro inflammation assay.

Target Gene		Primers
18S	Forward	GTAACCCGTTGAACCCCATT
	Reversed	CCATCCAATCGGTAGTAGCG
KDM5D	Forward	TAACACACACCCGTTTGACAA
	Reversed	GCTGCTGAACTTTGAAGGCTG
IL-1b	Forward	5′-GCTGAGGAAGATGCTGGTTC-3′
	Reversed	5′-TCCATATCCTGTCCCTGGAG-3′
COX2	Forward	5′-GCCCGACTCCCTTGGGTGTC-3′
	Reversed	5′-TTGGTGAAAGCTGGCCCTCGC-3′

KDM5D: lysine demethylase 5D; IL-1b: Interleukin 1 beta; COX2: prostaglandin-endoperoxide synthase 2 (PTGS2).

**Table 2:** Cell composition of tSVF based on CD marker expression.

Patient CD Markers	#1	#2	#3	#4	#5	#6	Cell Population
CD45−; CD90+; CD105+	32.90%	44.40%	73.70%	36.00%	59.90%	28.30%	Mesenchymal stromal cell
CD31+; CD34+	55.70%	57.50%	88.00%	17.00%	10.90%	8.20%	Endothelial cell
CD45+; CD34−	3.10%	0.70%	0.90%	5.80%	1.40%	8.60%	Leukocyte
CD34+/−; CD31−; CD146+	0.20%	0.20%	0.10%	0.40%	0.00%	0.50%	Pericyte
CD45+; CD34+	0.50%	0.00%	1.00%	0.00%	0.10%	0.30%	Hematopoietic stem cell-like cell
CD34bright; CD31−, CD146−	20.30%	12.50%	2.90%	0.00%	0.00%	0.00%	Supra-adventitial cell

## Data Availability

Still available upon asking the authors.

## Appendix A

**FAT procedure:** The lipoaspirate was harvested from the abdomen and upper legs with a lipo-harvesting cannula (Tulip, Medical Products, San Diego, CA, USA) after distribution of 500 mL modified Klein’s solution (per 500 mL saline, 20 mL of lidocaine, 1% epinephrine 1:100,000 and 2 mL of bicarbonate). The FAT procedure with a re-usable one-hole fractionator in a closed system was performed with the disposable Arthrex Autologous Conditioned Plasma (ACP) Double Syringe Systems (Arthrex, Inc. ABS-10014, Naples, Italy). Disposable ACP double syringes were filled with decanted lipoaspirate and centrifuged at 956× *g* for 2.5 min with a swing out rotor centrifuge at room temperature (RT) (Hettich Rotofix 32, benchtop, swing out rotor, Tuttlingen, Germany). After centrifugation, the upper oily fraction was discarded by pulling the inner syringe. Then, the lower aqueous fraction was discarded by opening the red closing cap yielding condensed lipoaspirate. Condensed lipoaspirate was used for mechanical dissociation according to the fractionation of adipose tissue (FAT) procedure. After mechanical dissociation, samples were centrifuged again for 2 min. at 760× *g*. This yielded an oily fraction that could be easily removed leaving tSVF in the larger outer ACP double syringe.
